# The effectiveness of community treatment orders (CTOS) across Birmingham and Solihull Mental Health Foundation Trust (BSMHFT)

**DOI:** 10.1192/bjo.2021.284

**Published:** 2021-06-18

**Authors:** Sambavi Navaratnarajah, Riya Basu, Syed Rashid

**Affiliations:** 1 BSMHFT; 2 North AOT, BSMHFT

## Abstract

**Aims:**

To ascertain if CTOs ensure that patients are effectively treated in the community and maintain stability in their mental health.

If a patient requires recall(+/- revocation) that this is done in a timely fashion in according to the 1983 Mental Health Act.

**Background:**

A number of studies have been inconclusive in determining the benefits of CTOs in reducing the re-admissions of “revolving door” patients In Assertive Outreach (AO). It is felt that CTOs have reduced readmission of patients due to the intensive input from community teams, decreased recall and subsequent revocation. Those admitted are thought to require fewer inpatient days. It is clear that many patients who require recall following non-engagement, non-compliance, will accept medication following RC review. However at present in BSMHFT patients can only be recalled if they are allocated a bed. Due to the national bed shortage, this has resulted in delays following decision to recall and thus early and effective treatment for patients. In this aspect it defeats the role of the CTO as per the 2007 MHA.

**Method:**

CTO data from 1st April 2018 to 31st March 2019 was obtained from all 6 AOT's in BSMHFT. The following factors were considered;

1. Time between decision to recall by RC and recall to inpatient facility
2. Number of recalls converted to revocations
3. Number of inpatient bed days if revoked
4. Number of admissions on CTO
5. Patient/family agreement of CTO

**Result:**

98 CTO patients were recorded over this period. 19 out of 26 recalls had recall dates documented. 10 recalls were revoked due to relapse of mental illness. Average days from RC recall decision to actual recall or cancellation was 63.89 days. Main reasons for delay were bed unavailability and execution of warrant.

Following revocation, average inpatient bed days was 103.71. 41% of families agreed with CTOs, 36% of patients contested their CTO.

**Conclusion:**

Over a quarter of patients on CTO were recalled to hospital however, less than half of these had their CTO revoked. The remainder accepted treatment following urgent community review whilst on the bed list. Evidently the majority of patients didn't need admission. With the ongoing bed crisis, alternative avenues need to be sought to ensure prompt treatment and prevent relapse.

A CTO suite designed for recall could be the solution for the future.

